# Gender-specific dual effects of physical activity on depression and mortality: a nine-year cohort study in Chinese adults aged 45 and above

**DOI:** 10.3389/fpubh.2025.1510044

**Published:** 2025-01-21

**Authors:** Dan Shan, Meina Yang, Kunyan Zhou

**Affiliations:** ^1^Department of Obstetrics and Gynecology, West China Second University Hospital, Sichuan University, Chengdu, China; ^2^Key Laboratory of Birth Defects and Related Diseases of Women and Children (Sichuan University), Ministry of Education, Chengdu, China

**Keywords:** physical activity, depression, all-cause mortality, CHARLS, menopause

## Abstract

**Background:**

Regular participation in physical activity (PA) reduces all-cause mortality (ACM) in the general population. However, the effects of PA on depressed patients and potential gender-specific responses have not been fully elucidated. In this study, we aimed to investigate the role of PA on new-onset depression and ACM in Chinese adults aged 45 year and older, with particular emphasis on gender differences.

**Methods:**

This was a longitudinal cohort study that took place over a nine-year period and featured 2,264 participants drawn from the China Health and Retirement Longitudinal Study (CHARLS). PA levels were categorized into quartiles using metabolic equivalents (MET; minutes/week), and depression was evaluated according to the 10-item Center for Epidemiologic Studies Depression Scale (CESD-10) scale. Specific relationships between PA, depression, and mortality were then investigated by applying multivariate logistic regression and Cox proportional hazards models.

**Results:**

Highest quantile levels of PA were correlated with a 37% increase in the risk of new-onset depression in middle-aged (45–59 years) and older adults (>60 years). This association was predominantly influenced by a significant increase in the risk of mild depression (a score of 10–14 on the CESD-10) (odds ratio [OR]: 1.76; 95% confidence interval [CI]: 1.29–2.42, *p* < 0.001), with a more pronounced effect observed in women (OR: 1.83; 95% CI: 1.26–2.66, *p* = 0.002). A critical threshold for PA was identified at 4536 MET-minutes/week, beyond which the risk of depression increased significantly (*p* < 0.05). Conversely, higher levels of PA were linked to a 90% reduction in ACM (HR: 0.10; 95% CI: 0.02–0.44, *p* = 0.002), with the effect being more pronounced in men.

**Conclusion:**

While PA reduces mortality, excessive activity may increase the risk of mild depression, particularly in women. These findings highlight the need for gender-specific PA guidelines that balance physical and mental health outcomes.

## 1 Introduction

Depression is a prevalent mental disorder marked by enduring feelings of sadness and a sustained reduction in the capacity to experience pleasure or participate in physical activities (1). Globally, depression has become one of the leading causes of disability and reduced quality-of-life, particularly among the older adult, for which the incidence and burden of depression are steadily increasing (2). Contemporary estimates indicate that approximately 4.4% of the global population is afflicted by depression (2). Projections suggest that by 2030, depression will ascend to become the second leading cause of global disease burden (2–4). Furthermore, between 1990 and 2019, the global incidence of depression, as measured by disability-adjusted life years (DALYs), increased by 61.1% (5). Numerous studies on depression have been conducted in the older adult population of China who are 60 years of age or older. Since the 1990s and have demonstrated a high prevalence of depressive symptoms; however, due to regional differences and variations in measurement methods, the reported prevalence rates varied widely, ranging from 6.3 to 53.6% (6, 7). Some researchers believe that traditional Chinese family values, such as strong familial bonds and filial piety, have contributed to the relatively low rates of depressive symptoms among the older adult in China. However, these cultural factors are being eroded by socioeconomic transitions, an acceleration in the pace of life, and the rapid industrialization and urbanization; these factors have reduced family size and weakened family functions (for example, providing support, care, and guidance within a family), thus diminishing the protective effect of traditional culture against depression in the older adult (8, 9). Consequently, preventing and managing depression have become critical tasks for public health in China.

Physical activity (PA) is a crucial component of healthy aging. It has been shown to help prevent or reduce the risk of cardiovascular diseases, diabetes, cancer, sarcopenia, osteoporosis, cognitive impairment, and improve mental health (10, 11). However, these benefits may vary depending on gender, age, and the intensity and frequency of PA (12). Studies relating to the distribution of PA and strenuous labor in the older adult population have identified significant differences in the levels of PA among older adults in China (10, 12). Most older adult individuals generally have low levels of PA, making it difficult for them to meet recommended health standards; these low levels of PA are primarily due to the conveniences of modern life and the widespread use of communication technologies (12). However, older adults that inhabit rural areas, and those living under poor economic conditions or in strained relationships with their children, often still engage in high-intensity physical labor to sustain their livelihoods. In addition, there are other non-economic reasons underlying the need for high-intensity PA, including household chores, caring for grandchildren, socializing, or participating in sports activities (13). A range of biological factors (genetics and neuroplasticity), socioeconomic factors and lifestyle factors (including PA, nutrition, and the environment), are relatively controllable and known to be closely related to depression (14–21). Regular PA has been shown to promote neurogenesis and improve mood-regulating neurotransmitters like dopamine and serotonin, while the specific impact of PA on mental health in older adults remains underexplored (22). China, home to almost one-fifth of the world’s population, is experiencing an increasing population of older adults. As such, investigating the specific relationship between controllable risk factors, such as PA, and depression is of significant public health importance for managing the health of middle-aged and older adults.

Although several studies have investigated the relationship between PA and depression, most of the previous studies focused on adolescent or young adult populations and were mostly cross-sectional or small randomized controlled trials (23). Consequently, there is a significant lack of clarity regarding the specific effect of long-term PA patterns on depression in middle-aged and older populations. U.S cohort study shows that women compared with men derived greater gains in cardiovascular disease risk reduction from equivalent doses of leisure-time PA (24, 25). In addition, there may be significant differences in the impact of PA on males and females, although there is currently extremely limited research findings related to gender differences (25). The China Health and Retirement Longitudinal Study (CHARLS) is a prospective cohort study that covers almost all provinces in mainland China. In this study, we used data from the CHARLS and investigated the association between PA levels and the risk of new-onset depression and all-cause mortality (ACM) over a nine-year period, with specific emphasis on gender differences. By analyzing data from 2,264 participants (aged 45 years and older), we sought to identify critical thresholds for PA and provide evidence for the future development of personalized guidelines. Given China’s rapidly aging population, our findings are likely to have important implications for public health policy, providing valuable insights for clinicians and policymakers to optimize health outcomes *via* tailored PA recommendations.

## 2 Methods

### 2.1 Study design and participants

**Figure 1:**
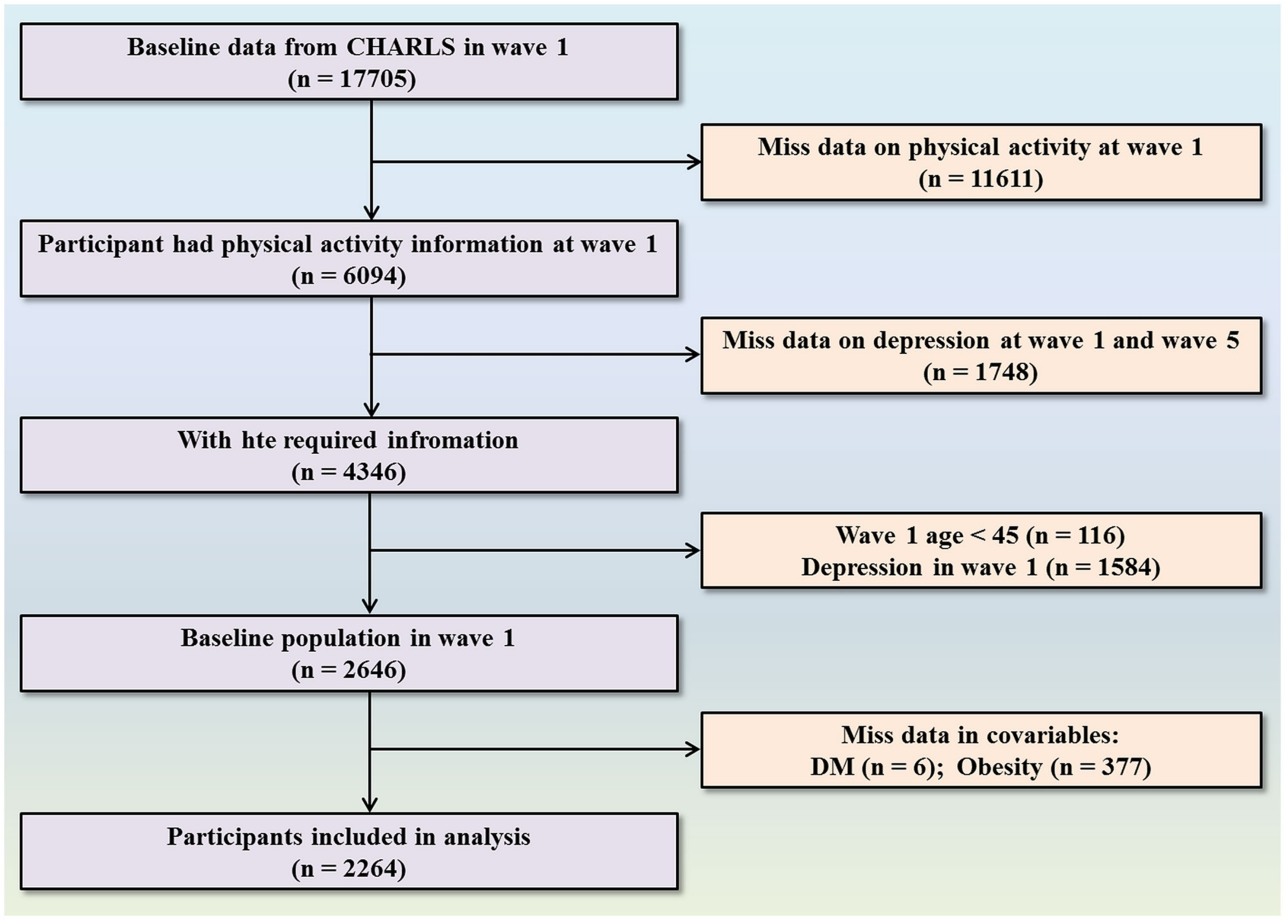
Flowchart of participant selection.

The research analyzed data acquired by the China Health and Retirement Longitudinal Study (CHARLS), a population-based, longitudinal, and prospective cohort study designed to evaluate the health and socioeconomic conditions of Chinese adults aged 45 years and older (26). THE CHARLS employs stratified sampling and probability-proportional-to-size (PPS) sampling techniques, and covers 150 district-level units and 450 village-level units within communities or villages across 126 cities and counties in 28 provinces of China. Consequently, the CHARLS dataset is highly representative and provides an accurate reflection of the overall conditions of the older adult population in both urban and rural areas throughout China (27). The initial baseline assessment was administered in 2011 (wave 1), followed by subsequent follow-up assessments at intervals of 2–3 years. Thus far, five waves of data collection have been completed by the CHARLS, encompassing 2011, 2013, 2015, 2018, and 2020. Individuals who participated in the CHARLS program have all provided written and informed consent. In addition, the CHARLS program follows the Declaration of Helsinki’s ethical guidelines and was approved by the Ethics Review Committee of Peking University (Reference number: IRB00001052–11015). Further details relating to the CHARLS cohort have been described previously (27, 28). We performed a nine-year longitudinal cohort study, covering the period between 2011 and 2020, and used 2011 as the baseline for population data. We included all participants with complete PA records. Individuals with a diagnosis of depression at baseline or lacking follow-up depression data were excluded. We followed our study cohort from 2011 to 2020. Of the 17,705 participants that were initially evaluated, 15,441 were excluded due to missing age information, an age below 45 years (*n* = 116), insufficient PA data (*n* = 11,611), incomplete depression data at both wave 1 and wave 5 (*n* = 1,748), or an established diagnosis of depression at baseline (*n* = 1,584). Consequently, our final analysis involved 2,264 participants, including 1,584 without depression and 680 who were diagnosed with new-onset depression during the follow-up period (Figure 1).

### 2.2 Assessment of PA

Participants used a standardized questionnaire to self-report their PA, detailing intensity levels (vigorous, moderate, or walking), the amount of time spent performing PA (categorized as over 4 h, 2–4 h, 30 min to 2 h, and under 30 min), and how often they engaged in these activities (1–7 days per week). This questionnaire, incorporated within the CHARLS survey (pages 70–71), was methodologically aligned with the abbreviated version of the International Physical Activity Questionnaire (IPAQ), an internationally acknowledged instrument for the collection of PA data (29). There are three main distinctions between the CHARLS and IPAQ in terms of assessing PA: the reference period, the inclusion of sedentary behavior data, and the method of recording time. However, previous studies have shown that these differences do not significantly impact the assessment results, and the validity of the IPAQ has been confirmed in older adults (13, 30). In the present study, PA was calculated using metabolic equivalents (METs) using the specific formulae provided in Supplementary Table S1 (13, 26, 29, 30).

### 2.3 Assessment of depression

The CHARLS study applies the 10-item Center for Epidemiologic Studies Depression Scale (CES-D-10) to evaluate depressive symptoms. This instrument has been validated for both middle-aged and older adult Chinese populations (31, 32). The brief version of CESD-10 consists of 10 items (being bothered by things, having trouble keeping mind, feeling depressed, feeling that everything is an effort, feeling hopeful about the future, feeling fearful, restless sleeping, being happy, feeling lonely, inability to get going), each focusing on various aspects of negative emotions. The scale evaluates mood frequency using a rating of 0–3, with positive questions scored in reverse. The CESD-10 items were incorporated into the CHARLS questionnaire and data were collected by computer-assisted interviews. The scores from all items were summed to create a depression score ranging from 0 to 30, with higher scores indicating more severe depressive symptoms (33). Participants were categorized as having depression if their CESD-10 score was 10 or above (34). The severity of depression was categorized into four levels: scores of 0 to 9 were considered normal, 10 to 14 indicated mild depression, 15–19 represented moderate depression, and scores of 20 and above were classified as severe depression.

### 2.4 Assessment of all-cause death

Data relating to ACM were available during the 2020 follow-up survey (wave 5). There were 6,094 participants in wave 1 with information related to PA; of these 1,080 were lost to follow-up, 124 were excluded because they were aged <45 years, 1,863 were excluded because they had depression in wave 1, and 534 were excluded due to an incomplete dataset. Finally, 2,493 participants (2,442 alive and 51 dead) were included in our cohort to evaluate the relationship between PA and ACM over 9 years of follow-up.

### 2.5 Covariates

We selected covariates based on prior research (33, 35). To evaluate the independent effect of PA on depression, we incorporated several covariates, including sociodemographic factors, including age, gender, and marital status (categorized as married, divorced, or never married); residence (rural or urban); and education level (high school or above, high school or below, or illiterate). Marital status was further detailed into ‘married’ (including those temporarily separated due to work), ‘divorced’ (including widowed and separated individuals), and ‘never married,’ Lifestyle factors such as smoking status and alcohol consumption were also considered, with drinking frequency classified as ‘never,’ ‘<1 time/month,’ and ‘≥1 time/month’. We also considered health status, including current diseases such as hypertension (defined by a systolic blood pressure ≥ 140 mmHg and a diastolic blood pressure ≥ 90 mmHg) self-reported hypertension, or the use of blood pressure-lowering medications, and diabetes. Body weight status was included as a covariate, categorized by body mass index (BMI) into low weight, normal weight, overweight, and obesity. Since the general retirement age in China is 60 years old and the average life expectancy is around 70 years old (36), participants were divided into age groups of 45–60 years, 60–70 years, and over 70 years, and all data were collected by trained investigators using standardized questionnaires.

### 2.6 Statistical analysis

Continuous variables are expressed as mean ± standard deviation (SD) or as medians with interquartile range (IQR), whereas categorical variables are reported as frequencies and percentages. To investigate the differences in baseline characteristics across groups, we used the Student’s *t-*test or Mann–Whitney *U* test for continuous variables and the Chi-squared test or Fisher’s exact test for categorical variables, thereby ensuring appropriate group comparisons. We categorized PA into quartiles as metabolic equivalents (METQ) variables (Q1: 49.5–2446.5, Q2: 2446.5–6,720, Q3: 6720–13,440, Q4: 13440–25,704); the lowest quartile was used as the reference category.

Trend testing was performed to evaluate whether different levels of PA showed a systematic, ordered relationship with depression. Next, we performed multivariate logistic regression analyses to investigate the relationship between PA and the risk of developing depression. Odds ratios (ORs) with 95% confidence intervals (CIs) were calculated using three distinct logistic models: Model 1 was a crude model with no adjustments; Model 2 adjusted for sociodemographic variables, including age, education, marital status, and residence, to reduce the interference of these social factors on physical activity and depression; and Model 3 further adjusted for additional confounders such as smoking status, alcohol consumption, obesity, hypertension, and diabetes mellitus (DM), to reduce the limitations on physical activity caused by unhealthy lifestyle habits and comorbidities, or depression induced by underlying diseases. We also performed subgroup analyses to investigate the association between PA and depression across a range of demographic and clinical characteristics, including age, sex, marital status, smoking, drinking, hypertension, diabetes, and obesity. These analyses were adjusted for all covariates, except the stratification factor itself. In addition, interaction effects were evaluated to determine whether the relationship between PA and depression differed according to subgroup variables, thereby identifying potential moderators of this association.

To examine the relationship between PA and varying levels of depression severity (mild, moderate, severe), we employed a multinomial logistic regression model, designating PA as the independent variable and depression severity as the dependent variable. Furthermore, we utilized restricted cubic splines (RCS) to visually represent the dose–response relationship between sleep duration and the risk of developing depression, with knots strategically placed at the 5th, 35th, 65th, and 95th percentiles. We conducted two sensitivity analyses. First, we replicated the primary analyses by utilizing the complete dataset (*N* = 2,464) with imputed missing covariates. Second, we performed the analyses but excluded participants aged 70 and above due to their lower likelihood of engaging in vigorous PA. The effect of PA on ACM was evaluated by applying a multivariable Cox proportional hazards regression model, employing three models analogous to those used in the logistic regression analysis. Hazard ratios (HRs) with 95% confidence intervals (CIs) were reported. All statistical analyses were performed using R software (Version 4.4.1; R Foundation for Statistical Computing, Vienna, Austria; available at https://www.r-project.org), with a significance level set at *p* < 0.05 for two-tailed tests.

## 3 Results

### 3.1 Population characteristics

Table 1 provides a summary of the characteristics of the study participants. The final cohort featured 2,264 individuals; of these, 680 participants were newly diagnosed with depression. The median age of the participants was 56 years; 49.25% were males (*n* = 1,115) and 50.75% were females (*n* = 1,149). Comparative analysis revealed that participants with new-onset depression were more significantly likely to be female, possess lower educational attainment, reside in rural areas, be non-smokers, non-drinkers, free from hypertension or diabetes, and maintain a normal weight, in comparison to those without depression (*p* < 0.05). The disparity between rural and urban areas is evident across different levels of physical activity, particularly in high-intensity activities. In the Q3 and Q4 groups, the proportion of physical activity participants in rural areas is significantly higher than in urban areas, with rural participation rates of 69.1 and 79.3%, compared to only 30.9 and 20.7% in urban areas (Supplementary Table S2).

**Table 1:** Baseline characteristics of study population by depression status at follow-up.

Variable	Total (*n* = 2,264)	Normal (*n* = 1,584)	Depression (*n* = 680)	*p*
Age (mean ± SD)	56.76 ± 7.91	56.66 ± 7.95	56.99 ± 7.81	0.35
Age group, *n* (%)				0.69
45–60	1,481 (65.42)	1,045 (65.97)	436 (64.12)	
60–70	622 (27.47)	429 (27.08)	193 (28.38)	
>70	161 (7.11)	110 (6.94)	51 (7.50)	
Sex, *n* (%)				**<0.0001**
Female	1,149 (50.75)	739 (46.65)	410 (60.29)	
Male	1,115 (49.25)	845 (53.35)	270 (39.71)	
Marital, *n* (%)				0.24
Divorced	161 (7.11)	114 (7.20)	47 (6.91)	
Married	2094 (92.49)	1,466 (92.55)	628 (92.35)	
Never married	9 (0.40)	4 (0.25)	5 (0.74)	
Education, *n* (%)				**<0.0001**
Illiterate	501 (22.13)	302 (19.07)	199 (29.26)	
High school or below	1,501 (66.30)	1,062 (67.05)	439 (64.56)	
High school or above	262 (11.57)	220 (13.89)	42 (6.18)	
Residence, *n* (%)				**<0.0001**
Rural	1,417 (62.59)	916 (57.83)	501 (73.68)	
Urban	847 (37.41)	668 (42.17)	179 (26.32)	
Smoke, *n* (%)				**<0.0001**
Never smoke	1,402 (61.93)	934 (58.96)	468 (68.82)	
Former, now quit	164 (7.24)	129 (8.14)	35 (5.15)	
Still smoke	698 (30.83)	521 (32.89)	177 (26.03)	
Drink, *n* (%)				**<0.001**
<1 time/month	187 (8.26)	136 (8.59)	51 (7.50)	
≥1 time/month	614 (27.12)	464 (29.29)	150 (22.06)	
No	1,463 (64.62)	984 (62.12)	479 (70.44)	
Obesity, *n* (%)				0.24
Low weight	82 (3.62)	50 (3.16)	32 (4.71)	
Normal	1,186 (52.39)	830 (52.40)	356 (52.35)	
Obesity	262 (11.57)	191 (12.06)	71 (10.44)	
Overweight	734 (32.42)	513 (32.39)	221 (32.50)	
Hypertension, *n* (%)				0.92
No	1,460 (64.49)	1,023 (64.58)	437 (64.26)	
Yes	804 (35.51)	561 (35.42)	243 (35.74)	
DM, *n* (%)				0.66
No	2033 (89.80)	1,419 (89.58)	614 (90.29)	
Yes	231 (10.20)	165 (10.42)	66 (9.71)	
PA, *n* (%)				**<0.0001**
Q1	567 (25.04)	413 (26.07)	154 (22.65)	
Q2	565 (24.96)	424 (26.77)	141 (20.74)	
Q3	566 (25.00)	388 (24.49)	178 (26.18)	
Q4	566 (25.00)	359 (22.66)	207 (30.44)	

Bold *p* values represent *p* < 0.01.

### 3.2 Longitudinal association between PA and the risk of new-onset depression

Multivariate regression analyses were performed by stratifying PA into quartiles. The Q4 group showed a 37% increase in the risk of new-onset depression when compared to the Q1 group (OR: 1.37; 95% CI: 1.05–1.81). Trend tests revealed a significant dose–response relationship, indicating that as the levels of PA increased, so did the risk of developing depression (*p* for trend <0.05, Table 2). Gender-specific analysis revealed distinct patterns. When considering males, levels of PA did not significantly influence the risk of depression across all quartiles. In model 2, which was adjusted for all covariables, the OR for Q4 was 1.04 (95% CI: 0.69–1.57, *p* = 0.86), showing no significant increase in risk (Table 2). In contrast, females in the Q4 group exhibited a two-fold higher risk of developing depression when compared to those in Q1 (OR: 2.23; 95% CI: 1.57–3.16, *p* < 0.0001); furthermore, this association remained strong after adjustment in Model 2 (OR: 1.83; 95% CI: 1.26–2.66, *p* = 0.002). Trend analysis further confirmed a significant dose–response relationship in women, with higher levels of PA correlating with an increased risk of depression (*p* for trend <0.001). These findings highlight a notable difference between genders, with higher levels of PA being significantly associated with an increased risk of depression in women (Table 2).

**Table 2:** Prospective associations between baseline PA with follow-up new-onset depression in CHARLS.

PA	Crude model		Model 1		Model 2	
	OR (95%CI)	*p*	OR (95%CI)	*p*	OR (95%CI)	*p*
METQ		**<0.0001**		**0.03**		**0.01**
Q1	ref		ref		ref	
Q2	0.89 (0.68, 1.16)	0.40	0.83 (0.63, 1.09)	0.18	0.85 (0.65, 1.12)	0.26
Q3	1.23 (0.95, 1.59)	0.11	1.07 (0.82, 1.39)	0.63	1.11 (0.85, 1.45)	0.45
Q4	1.55 (1.20, 1.99)	**<0.001**	1.25 (0.96, 1.63)	0.10	1.37 (1.05, 1.81)	**0.02**
Q1	ref		ref		ref	
Q2	0.72 (0.47, 1.11)	0.13	0.66 (0.43, 1.03)	0.07	0.65 (0.42, 1.02)	0.06
Q3	1.05 (0.70, 1.59)	0.80	0.91 (0.59, 1.39)	0.66	0.89 (0.58, 1.36)	0.59
Q4	1.29 (0.88, 1.88)	0.20	1.06 (0.71, 1.59)	0.77	1.04 (0.69, 1.57)	0.86
Female METQ		**<0.0001**		**0.001**		**<0.001**
Q1	ref		ref		ref	
Q2	1.08 (0.76, 1.51)	0.68	1.01 (0.72, 1.44)	0.93	1.01 (0.71, 0.43)	0.97
Q3	1.43 (1.03, 2.00)	**0.03**	1.28 (0.91, 1.81)	0.16	1.29 (0.91, 1.83)	0.15
Q4	2.23 (1.57, 3.16)	**<0.0001**	1.8 (1.24, 2.60)	**0.002**	1.83 (1.26, 2.66)	**0.002**

Crude model: adjust for non-variables.

Model 1: adjust for age, education, marital, and residence.

Model 2: adjust for age, education, marital, residence, DM, obesity, hypertension, smoke, drink.

Bold *p* values represent *p* < 0.01.

**Figure 2:**
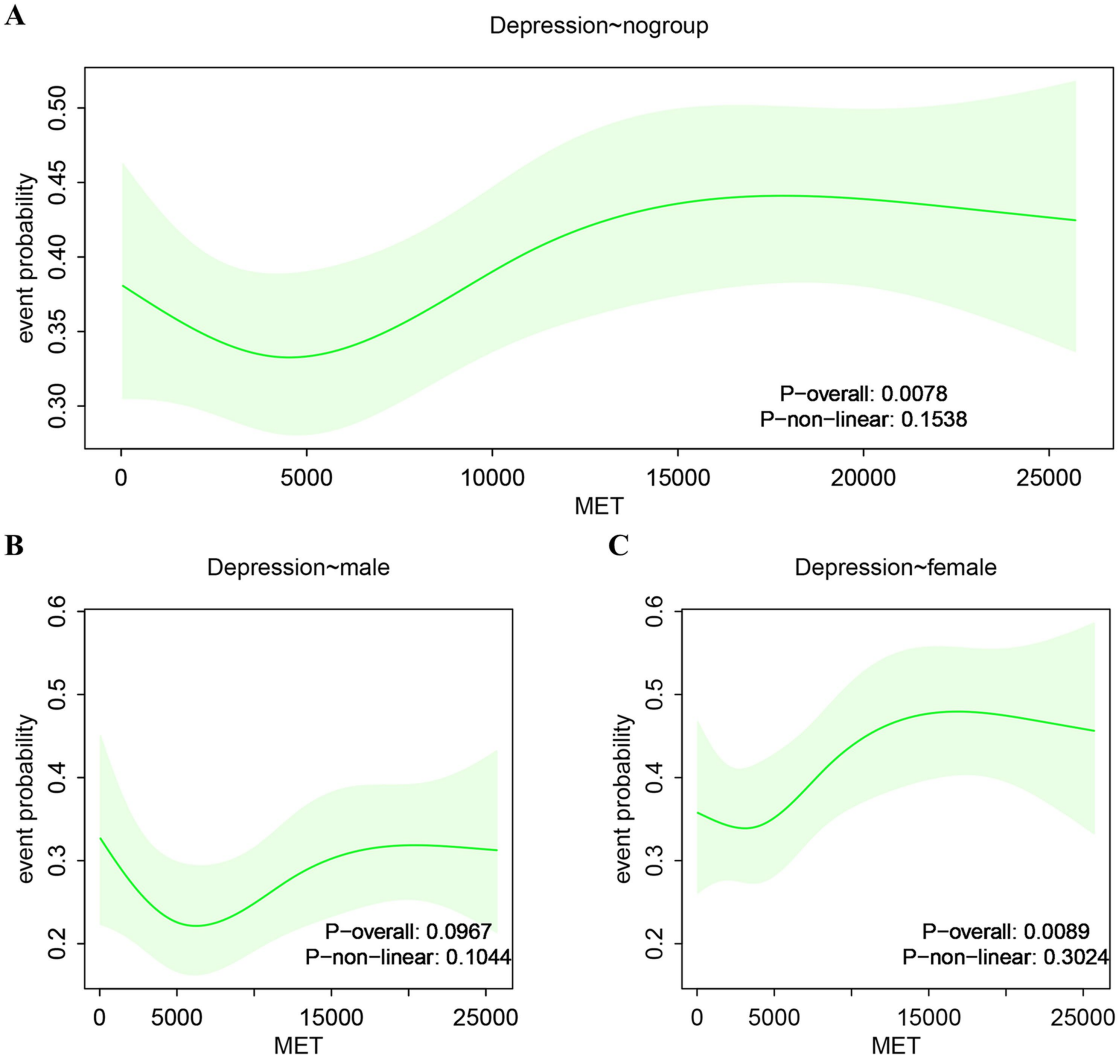
Restricted cubic splines analysis. **(A)** Association between PA and new-onset depression. **(B)** Association between PA and male group depression. **(C)** Association between PA and female group depression.

The RCS analysis generated an ‘S-shaped’ curve relating to the association between PA and new-onset depression (Figure 2), with a particular focus on gender differences. Although our analysis did not identify a significant non-linear relationship (*p* non-linear = 0.153), a notable inflection point was identified at approximately 4,536 MET-minutes/week; beyond this inflection point, the risk of depression increased significantly (*p* < 0.05). In males, there was no significant association between the levels of PA and the risk of depression (Figure 2B), thus indicating that increased PA did not influence the risk of new-onset depression in men. However, a different pattern was evident in females (Figure 2C); women exhibited a higher risk of new-onset depression when compared to men at the same level of PA. Overall analysis revealed an increasing trend in the risk of depression with higher levels of PA (Figure 2A), driven primarily by the female subgroup. These findings highlight the differential impact of PA on depression across the two genders, indicating that women are more susceptible to experiencing negative mental health outcomes associated with high levels of PA.

Further analysis, identified a significant variation in the relationship between baseline PA and differing degrees of depression (Table 3). Compared to the Q1 group, participants in the Q4 group had a significantly higher risk of mild depression (OR = 1.769, 95% CI: 1.291–2.424, *p* < 0.001), while the risks for moderate and severe depression were not significantly different (OR = 1.217, 95% CI: 0.812–1.824, *p* = 0.34; OR = 1.517, 95% CI: 0.856–2.687, *p* = 0.15, respectively). These findings suggest that PA may differentially impact the severity of depression, demonstrating that higher levels of PA could represent a significant risk factor for mild depression.

**Table 3:** Prospective associations between baseline PA with follow-up new-onset different degree depression in CHARLS.

PA	Mild depression		Moderate depression		Vigorous depression	
	OR (95% CI)	*p*	OR (95% CI)	*p*	OR (95% CI)	*p*
Q1	ref	ref	ref	ref	ref	ref
Q2	0.91 (0.64, 1.28)	0.60	0.86 (0.56, 1.31)	0.48	0.88 (0.47, 0.64)	0.70
Q3	1.49 (1.08, 2.04)	**0.01**	0.94 (0.61, 1.43)	0.77	0.96 (0.52, 1.80)	0.91
Q4	1.76 (1.29, 1.42)	**<0.001**	1.21 (0.81, 1.82)	0.34	1.51 (0.85, 2.68)	0.15

Bold *p* values represent *p* < 0.01.

### 3.3 Subgroup analyses

Subgroup analyses identified significant differences in the association between PA and the risk of new-onset depression across various populations. When considering individuals aged 45–60 years, those in the highest quartile of PA (Q4) had a significantly higher risk of developing depression when compared to those in the lowest quartile (Q1) (OR = 1.73, 95% CI: 1.22–2.47, *p* < 0.001). Gender analysis further revealed that women in the Q4 group had a significantly increased risk of depression when compared to those in the Q1 group (OR = 1.88, 95% CI: 1.30–2.73, *p* < 0.001). No significant association was observed in men. Furthermore, when considering participants residing in urban regions, those in the Q4 group exhibited a significantly higher risk of depression (OR = 2.24, 95% CI: 1.33–3.76, *p* = 0.01). Non-smokers and non-drinkers in the Q4 group also had an elevated risk of depression (OR = 1.57, 95% CI: 1.12–2.21, *p* = 0.005; OR = 1.68, 95% CI: 1.21–2.33, *p* = 0.001, respectively). These findings suggest that the relationship between PA and the risk of depression varied significantly across different subgroups, particularly among specific age groups, genders, and lifestyle habits (Table 4). We also used subgroup analyses to evaluate the interaction between PA and the risk of new-onset depression across different demographic and clinical characteristics. While variations in the impact of PA on the risk of depression were observed across different subgroups, interaction analysis revealed that these differences were not statistically significant (*p* > 0.05; Table 4).

**Table 4:** Subgroup analysis of the association of PA and new-onset risk of depression.

Character	Q1	Q2	Q3	Q4	*p*
Age					0.44
45–60	ref	1.06 (0.74, 1.52)	1.33 (0.94, 1.89)	1.73 (1.22, 2.47)	**<0.001**
60–70	ref	0.77 (0.47, 1.26)	0.99 (0.61, 1.61)	1.29 (0.78, 2.12)	0.29
>70	ref	0.37 (0.14, 0.90)	0.57 (0.19, 1.59)	0.90 (0.29, 2.81)	0.7
Sex					0.29
Male	ref	0.65 (0.41, 1.00)	0.85 (0.56, 1.30)	1.00 (0.67, 1.50)	0.5
Female	ref	1.01 (0.71, 1.43)	1.28 (0.91, 1.81)	1.88 (1.30, 2.73)	**<0.001**
Residence					0.34
Urban	ref	1.05 (0.68, 1.62)	1.20 (0.75, 1.91)	2.24 (1.33, 3.76)	**0.01**
Rural	ref	0.74 (0.52, 1.06)	1.01 (0.73, 1.41)	1.19 (0.86, 1.65)	0.06
Smoke					0.81
Never smoke	ref	0.96 (0.69, 1.32)	1.20 (0.87, 1.65)	1.57 (1.12, 2.21)	**0.005**
Former, now quit	ref	0.57 (0.18, 1.68)	0.97 (0.29, 3.24)	0.68 (0.19, 2.26)	0.79
Still smoke	ref	0.69 (0.38, 1.25)	0.92 (0.54, 1.61)	1.27 (0.76, 2.14)	0.12
Drink					0.19
No	ref	0.84 (0.61, 1.15)	1.10 (0.81, 1.51)	1.68 (1.21, 2.33)	**0.001**
≥1 time/month	ref	0.86 (0.47, 1.60)	1.13 (0.63, 2.05)	0.83 (0.47, 1.50)	0.7
<1 time/month	ref	0.89 (0.28, 2.85)	0.77 (0.24, 2.50)	1.91 (0.68, 5.65)	0.16
DM					0.92
No	ref	0.88 (0.66, 1.17)	1.13 (0.85, 1.49)	1.47 (1.11, 1.95)	**0.002**
Yes	ref	0.88 (0.35, 2.18)	1.09 (0.47, 2.56)	1.55 (0.61, 4.00)	0.33
Hypertension					0.84
Yes	ref	0.79 (0.50, 1.24)	1.15 (0.74, 1.78)	1.48 (0.95, 2.33)	**0.04**
No	ref	0.92 (0.65, 1.30)	1.09 (0.78, 1.53)	1.42 (1.01, 2.00)	**0.02**
Obesity					0.6
Overweight	ref	0.95 (0.60, 1.49)	1.20 (0.76, 1.91)	1.59 (0.98, 2.59)	**0.04**
Obesity	ref	1.77 (0.82, 3.86)	1.48 (0.64, 3.43)	2.66 (1.02, 7.04)	**0.08**
Normal	ref	0.65 (0.43, 0.97)	1.04 (0.72, 1.51)	1.23 (0.85, 1.78)	**0.06**
Low weight	ref	0.55 (0.09, 3.03)	0.35 (0.06, 1.73)	0.64 (0.13, 3.08)	0.65

Adjusted for age, sex, marital status, education, smoking, drinking, hypertension, and DM, except for the stratification factor itself.

Bold *p* values represent *p* < 0.01.

### 3.4 Longitudinal association between PA and ACM

Table 5 presents the longitudinal analysis of PA and ACM over a nine-year period of follow-up. Higher levels of PA were inversely associated with ACM. In the crude model, participants in the highest quartile (Q4) had a significantly lower mortality risk when compared to those in the lowest quartile (Q1) (HR = 0.08, 95% CI: 0.02–0.32, *p* < 0.001). This association remained robust after adjusting for confounders (HR = 0.10, 95% CI: 0.02–0.44, *p* = 0.002). A consistent linear trend was observed across all models (*p* for trend <0.001). Gender-specific analysis further demonstrated that this protective effect was more pronounced in men (HR = 0.11, 95% CI: 0.02–0.48, *p* = 0.003), while the association was not significant in women (HR = 0.89, 95% CI: 0.53–4.28, *p* = 0.74). Collectively, these findings suggest that higher levels of PA are significantly associated with reduced ACM risk in men.

**Table 5:** Hazard ratio of PA and ACM among cohort participants follow-up 9 years.

PA	Crude model		Model 1		Model 2	
	HR (95%CI)	*p*	HR (95%CI)	*p*	HR (95%CI)	*p*
METQ		**<0.0001**		**0.004**		**<0.001**
Q1	ref		ref		ref	
Q2	0.44 (0.22, 0.88)	**0.02**	0.54 (0.27, 1.07)	0.08	0.5 (0.25, 0.99)	0.05
Q3	0.43 (0.21, 0.87)	**0.02**	0.68 (0.32, 1.44)	0.32	0.64 (0.30, 1.35)	0.24
Q4	0.08 (0.02, 0.32)	**<0.001**	0.13 (0.03, 0.56)	**0.01**	0.1 (0.02, 0.44)	**0.002**
Male METQ		**<0.0001**		**0.002**		**0.002**
Q1	ref		ref		ref	
Q2	0.32 (0.13, 0.76)	**0.01**	0.34 (0.14, 0.82)	**0.02**	0.34 (0.14, 0.81)	**0.02**
Q3	0.4 (0.18, 0.91)	**0.03**	0.57 (0.24, 1.35)	0.20	0.56 (0.23, 1.35)	0.20
Q4	0.08 (0.02, 0.32)	**<0.001**	0.11 (0.03, 0.51)	**0.005**	0.11 (0.02, 0.48)	**0.003**
Female METQ		**0.03**		0.34		0.4
Q1	ref		ref		ref	
Q2	0.75 (0.24, 2.37)	0.63	1.11 (0.35, 3.58)	0.86	1.42 (0.41, 4.88)	0.58
Q3	0.46 (0.12, 1.79)	0.26	0.97 (0.23, 4.10)	0.97	0.94 (0.22, 4.04)	0.94
Q4	0.55 (0.53, 1.19)	0.72	0.90 (0.57, 9.66)	0.71	0.89 (0.53, 4.28)	0.74

Crude model: adjust for none.

Model 1: adjust for age, education, marital, and residence.

Model 2: adjust for age, education, marital, residence, DM, obesity, hypertension, smoke, drink.

Bold *p* values represent *p* < 0.01.

### 3.5 Sensitivity analyses

Finally, sensitivity analyses were performed to evaluate the robustness of the association between PA and the risk of new-onset depression (Table 6). First, we repeated the primary analysis using the complete dataset of 2,464 participants (1,860 without depression, 786 with depression), with missing covariables (*n* = 383) imputed. The outcomes were consistent with the primary analysis, demonstrating that participants in the highest quartile of PA (Q4) continued to have an increased risk of new-onset depression compared to those in the lowest quartile (Q1) (OR = 1.44, 95% CI: 1.11–1.86, *p* = 0.01). Secondly, we performed an additional analysis in which we excluded participants aged 70 years and older (*n* = 321) and those with an incomplete dataset (*n* = 346), leaving a final cohort of 2,103 participants (1,474 without depression, 629 with depression). This analysis also confirmed the stability of our findings, with the Q4 group showing a significantly higher risk of depression (OR = 1.47, 95% CI: 1.10–1.95, *p* = 0.01) compared to the Q1 group.

**Table 6:** Sensitivity analysis: association of PA and depression.

PA METQ	Crude model		Model 1		Model 2	
	OR (95%CI)	*p*	OR (95%CI)	*p*	OR (95%CI)	*p*
Miss input		**<0.0001**		**0.01**		**0.002**
Q1	ref		ref		ref	
Q2	0.96 (0.75, 1.22)	0.74	0.88 (0.69, 1.13)	0.32	0.9 (0.70, 1.15)	0.40
Q3	1.25 (0.98, 1.59)	0.07	1.06 (0.83, 1.36)	0.64	1.1 (0.85, 1.42)	0.45
Q4	1.63 (1.29, 2.06)	**<0.0001**	1.29 (1.01, 1.66)	**0.04**	1.44 (1.11, 1.86)	**0.01**
<70 years		**<0.0001**		**0.02**		**0.002**
Q1	ref		ref		ref	
Q2	0.99 (0.76, 1.31)	0.97	0.93 (0.70, 1.23)	0.61	0.96 (0.73, 1.28)	0.79
Q3	1.33 (1.02, 1.74)	0.04	1.16 (0.88, 1.53)	0.31	1.22 (0.92, 1.61)	0.17
Q4	1.62 (1.25, 2.11)	**<0.001**	1.32 (1.00, 1.74)	0.05	1.47 (1.10, 1.95)	**0.01**

Crude model: adjust for none.

Model 1: adjust for age, education, marital, residence.

Model 2: adjust for age, education, marital, residence, DM, obesity, hypertension, smoke, drink.

Bold *p* values represent *p* < 0.01.

## 4 Discussion

Our study found that higher levels of PA were associated with a 37% increased risk of new-onset depression, and that this risk increased by 83% in women. This association was consistent across various models. Higher levels of physical activity were found to significantly increase the risk of mild depression by 76%, but had no significant effect on moderate or severe depression. RCS analysis further identified an ‘S-shaped’ curve that demonstrated a sharp increase in the risk of new-onset depression when PA exceeded 4,536 MET-min/week. In addition, subgroup analysis indicated that women aged 45–60 years were particularly susceptible to developing depression if they did not smoke, drink alcohol, and did not have diabetes. Conversely, the highest quartile levels of PA were associated with a reduced risk of mortality, especially in men. Our study analyzed the dual impact of physical activity on depression and mortality, with a particular emphasis on gender differences. This underscores the importance of developing gender-specific, personalized physical activity strategies for health, providing evidence for the design of future public health policies targeting physical activity.

Our findings suggest that higher levels of physical activity are associated with an increased risk of new-onset depression, particularly in women. In addition, subgroup analysis revealed that women aged 45–60 years were particularly vulnerable to developing depression if they did not smoke, drink alcohol, or have diabetes. These findings appear to contradict the common perception that physical activity typically has a protective effect on mental health, while smoking, alcohol consumption, and diabetes are generally considered risk factors for various health issues (37). However, in China, women generally have lower rates of smoking and alcohol consumption (36). Additionally, PA is known to be a protective factor against diabetes (38) but might paradoxically contribute to a higher risk of depression in this demographic. Women who avoid these risk behaviors may be exposed to other significant depression risk factors, such as socioeconomic challenges or social isolation. One possible explanation is that, among Chinese women aged 45 and above, non-recreational activities such as household labor, child-rearing, and older adult care contribute significantly to their physical activity levels and may even constitute the primary component of their physical activity (39–41). However, these activities are often involuntary, non-recreational, and accompanied by substantial psychological stress, especially when engaging in high-intensity physical activity, which may trigger emotional distress and depressive symptoms. Therefore, high-intensity physical activity may amplify daily stressors, becoming a contributing factor to the increased risk of depression. Similarly, in this study, the prevalence of depression was significantly higher in rural areas compared to urban areas. Additionally, a higher proportion of rural residents engaged in high levels of physical activity than their urban counterparts. This suggests that the physical activities undertaken by rural residents may often be driven by survival pressures and necessity, associated with negative psychological and emotional factors, rather than for recreation or health purposes. Our study also found that high-intensity physical activity primarily increased the risk of mild depression, suggesting that high-intensity physical activity may exacerbate existing negative psychological backgrounds, thereby intensifying depressive symptoms rather than directly causing the onset of depression. Future research should explore the impact of physical activity within different psychological contexts to isolate the influence of psychological factors and more accurately understand the relationship between physical activity and depression.

The PA can influence depression through various pathways, including the nervous, immune and endocrine systems, and oxidative stress (42–45). Moreover, PA has been shown to modulate the immune response; while moderate exercise reduces inflammation, excessive activity may trigger an inflammatory response, thus contributing to depression (46). In this study, we unexpectedly identified a significant association between higher levels of PA and an increased risk of developing depression among middle-aged and older adults, with a 37% increase in the risk of depression. This finding contradicts the conclusions of several previous studies that generally support the protective effects of PA on mental health (47). These disparities may be attributed to differences in study design, population characteristics, and the methods used to measure PA. For instance, our study employed MET-minutes/week to quantify PA and categorized this parameter into four quartiles, thus providing a more detailed analysis of activity levels. In contrast, other studies may have applied only PA activity time or weekly frequency or different methodologies, potentially contributing to the varying outcomes observed. The beneficial effects of moderate PA on mental health, such as the reduction of anxiety and depressive symptoms, the enhancement of emotional stability, and the improvement of quality-of-life, have been extensively documented in the literature (47, 48). For example, a Mendelian randomization suggested that PA is a protective factor against depression (49). However, this previous study did not differentiate between different levels of PA; this omission could have obscured the potential risks associated with higher levels of exercise.

Our further analysis revealed that the increased risk of depression was predominantly linked to mild depression, with a 76% rise, while the association with moderate and severe depression was less pronounced. This observation may support a ‘moderate effect’ in which beneficial effects are achieved by performing moderate levels of PA. The RCS analysis we performed in our present study aligns with this concept, indicating that the risk of depression begins to rise notably at when the levels of activity exceed the 4,536 MET-min/week, a threshold that surpasses the World Health Organization’s (WHO) recommended levels 900–1,650 MET-min/week of PA for general health benefits (50, 51). Similarly, a previous systematic review and meta-analysis suggested that while 528 MET-min/week of PA can reduce the risk of depression by 25%, the benefits achieved by this activity may plateau or even diminish with higher levels of exposure, thus creating uncertainty with regards to the specific effects of excessive PA (52). Moreover, a previous longitudinal cohort study, conducted in Irish and featuring older adults (mean age: 61 years), found that frequent moderate-to-vigorous physical activity (MVPA) was significantly associated with a lower likelihood of depression. However, this previous study also reported that the minimum effective dose and the extent of additional protective effects beyond this level remain unclear (42). The findings of our study, and previous studies, offer a novel perspective on the complex and potentially bidirectional relationship between PA and depression.

Existing studies in China have reported that a range of factors are closely associated with higher levels of depressive symptoms, including age, retirement, physical disability, illness, a lack of social support (e.g., poor relationships with children), economic problems, life events, and low educational levels (28, 53). These findings suggest that both biological and socio-cultural factors play crucial roles in the heightened vulnerability to depression observed in perimenopausal and early postmenopausal women. In this study, we identified that women aged between 45 and 60 have a particularly high risk for the development of new-onset depression. Changes in estrogen levels and the pressures of living a multi-role life, among other things, not only increase women’s physical demands, but also create significant psychological stress (54–57). The lack of adequate leisure and recreation time may exacerbate this stress and fatigue, further increasing the risk of depression during this critical stage of life (58). In addition, we determined that elevated levels of PA led to a significant reduction in ACM, reducing the risk by 90%. Notably, an even more pronounced protective effect was observed in men, with a risk reduction of 89%. This finding aligns with the conclusions of numerous previous studies. For instance, a survey conducted in the United States demonstrated that relative to individuals who do not engage in physical exercise, the risk of ACM was reduced by 24% in women, and 15% in men, who regularly participated in PA. Engaging in 300 min of weekly exercise at moderate-intensity further reduced ACM by 19% in men and 14% in women (25). Other studies have demonstrated that intermittent moderate-to-vigorous PA significantly reduced the risk of cardiovascular disease in adults and extended lifespan (59). Furthermore, empirical evidence indicates that to achieve an optimal reduction in the risk of breast cancer, colon cancer, diabetes, ischemic heart disease, and ischemic stroke, an individual should engage in PA amounting to between 3,000 and 4,000 MET-minutes per week. This level of PA significantly surpasses the WHO recommendation of 600 MET-minutes per week. These data indicate that the benefits of PA may be optimized within a specific range (45). A key strength of this study, compared to previous research, is the exclusion of individuals with depression at baseline. When considering individuals with depression, some suffer from severe symptoms, including suicidal tendencies. In contrast, others often have comorbidities, such as cancer, stroke, disability, or the loss of function, which can significantly increase mortality risk. By excluding these individuals, our study demonstrated the pronounced protective effect of PA on ACM in a more specific manner.

The significant benefits of PA on ACM can be attributed to several key factors. First, it has been established that PA can improve cardiovascular health by reducing blood pressure, reducing the levels of low-density lipoprotein cholesterol (LDL-C), and by controlling obesity and diabetes, thereby reducing the risk of cardiovascular events which remain as a leading cause of ACM (60). In addition, a substantial body of epidemiological evidence supports the fact that individuals who engage in more PA are less likely to develop various cancers, thus reducing overall mortality (61, 62). For example, a previous study found that transportation activities, such as walking or cycling, could reduce the risk of breast cancer by 13%; in contrast, moderate and vigorous levels of PA could reduce the risk of breast cancer by 15 and 18%, respectively (11). Another study reported that moderate levels of PA could reduce the risk of lung cancer by 13%, while vigorous PA could reduce the risk by 30% (63). Our study addresses a gap in that previous research has generally identified PA as a protective factor for mortality but has not specifically examined gender differences in this relationship. However, in the present study, we identified a weaker, association between PA and mortality in women than in men, highlighting the importance of considering gender-specific responses in the evaluation of the protective effects of PA on longevity. This disparity may be related to the use of METs to quantify levels of PA without differentiating between various exercise intensities. The findings of our study reinforce the critical role of PA in reducing ACM, particularly in men, while also highlighting the importance of considering gender differences and individual needs in middle-aged and older women. Tailoring the intensity and type of PA to individual capabilities and needs could maximize health benefits for this population. Our findings suggest the importance of developing public health policies that promote gender-specific exercise guidelines and encourage tailored PA regimens based on individual capabilities and needs. This approach could enhance the effectiveness of interventions aimed at reducing mortality and improving overall health outcomes, particularly in middle-aged and older women.

### 4.1 Strengths and limitations

This study has several key strengths that need to be recognized. First, we utilized a substantial and representative sample from the CHARLS program with a nine-year longitudinal cohort design, thereby enhancing the generalizability of our findings and bolstering the credibility of the relationship between PA and the onset of depression. Second, PA data at baseline were collected by trained professionals, thus minimizing calculated bias and ensuring the reliability of the measurements recorded. Third, by investigating the relationships between PA, depression, and ACM, our study provides a comprehensive perspective on the health outcomes associated with PA. The novelty of our work lies in systematically analyzing these relationships across genders in a middle-aged and older adult population, thus challenging traditional health concepts and providing new insights for more precise and personalized health interventions. However, this study also has some limitations that need to be considered. First, as an ongoing study, the number of deaths was limited; this may have affected the precision of our analysis regarding the relationship between PA and ACM. Second, we did not collate data relating to the specific causes of death, thus limiting our ability to analyze the detailed health benefits and potential drawbacks of PA. Third, this study did not account for all potential confounding factors, such as the psychological context of physical activity and the purpose of the activity (whether for health and recreation or as passive household chores). Different psychological backgrounds can be important factors in the onset of depression. However, since this database does not include such information, we were unable to further analyze or discuss these factors, which also limits the causal inferences of this study. Future research should better control for these confounders to clarify the nature of this relationship. Fourth, our study found that individuals engaging in higher levels of physical activity were predominantly from rural areas, where the prevalence of depression is also higher compared to urban areas. The socio-cultural differences between rural and urban areas, such as family income, pension coverage, living environment, older adult care responsibilities, number of children, and caregiving for grandchildren, could be potential contributing factors to depression. However, this dataset does not provide such information. Future research should consider adjusting for these confounding factors to better clarify the impact of physical activity on depression. Finally, the self-reported PA and the CESD-10 scale used in this study may have introduced recall bias as the CESD-10 scale is primarily designed for screening depressive symptoms rather than for diagnosing clinical depression.

## 5 Conclusion

In this study, we identified a complex relationship between PA, depression, and ACM in middle-aged and older adults, emphasizing the critical role of both activity levels and gender. Collectively, our findings demonstrate that while moderate levels of PA significantly reduced ACM, particularly in men, higher levels of PA, exceeding 4,536 MET-minutes/week, were associated with an increased risk of new-onset depression, especially in women. This subtle dual effect suggests that we should consider the range of PA, gender differences, and other individualized characteristics, when developing guidance strategies for PA and health in middle-aged and older adults.

## Data Availability

The original contributions presented in the study are included in the article/Supplementary material, further inquiries can be directed to the corresponding author.
