# Supplementary material for: Gender-specific dual effects of physical activity on depression and mortality: a nine-year cohort study in Chinese adults aged 45 and above
Source: Front Public Health. 2025 Jan 21;13:1510044. doi: 10.3389/fpubh.2025.1510044 (PMC11791910; doi:10.3389/fpubh.2025.1510044)
Supplement: Supplementary file 1 [file Table_1.docx]

Supplementary Material

Table S1. PA calculated formulae.

| PA type (MET-minutes/week) | Formulae |
| --- | --- |
| Walking | 3.3 × walking minutes × walking days |
| Moderate | 4.0 × moderate-intensity activity minutes × moderate days |
| Vigorous | 0 × vigorous-intensity activity minutes × vigorous-intensity days |
| Total PA | Sum of walking + moderate + vigorous MET-minutes/week scores |

Table S2. Comparison of physical activity levels distribution between rural and urban areas.

| PA levels | Rual, n (%) | Urban, n (%) |
| --- | --- | --- |
| Q1 | 260 (45.9) | 307 (54.1) |
| Q2 | 317 (56.1) | 248 (43.9) |
| Q3 | 391 (69.1) | 175 (30.9) |
| Q4 | 449 (79.3) | 117 (20.7) |

PA, Physical activity.
